# Superlubricity of glycerol by self-sustained chemical polishing

**DOI:** 10.1038/s41598-019-42730-9

**Published:** 2019-04-18

**Authors:** Yun Long, Maria-Isabel De Barros Bouchet, Ton Lubrecht, Tasuku Onodera, Jean Michel Martin

**Affiliations:** 1 Université de Lyon, Ecole Centrale de Lyon, LTDS CNRS 5513, 69134 Ecully, France; 2 Université de Lyon, INSA de Lyon, LaMCoS, CNRS 5259, Villeurbanne, F69621 France; 3 Advanced Materials & Process Research Department, Center for Technology Innovation – Materials, Research & Development Group, Hitachi, Ltd., 7-1-1 Omika, Hitachi, 319-1292 Japan

**Keywords:** Environmental sciences, Chemistry

## Abstract

An impressive superlow coefficient of friction (CoF) as low as 0.004 (nearly equivalent to the rolling coefficient) was obtained by sliding a steel ball against a tetrahedral amorphous diamond-like carbon (ta-C) coating in glycerol under a boundary lubrication regime. X-ray photoelectron spectroscopy (XPS) and atomic force microscopy (AFM) revealed substantial changes in the surface chemistry and topography in the friction track. As shown by XPS analysis, a transfer of iron atoms from the steel ball to the ta-C layer occurred, forming iron oxy-hydroxide (FeOOH) termination on both surfaces. Between them, theoretical calculations show that a nanometre-thick fluid film consisting of glycerol and its degradation products prevents direct contact between the solid surfaces by nm-thick film EHL lubrication and results in the superlow friction, in agreement with the experiment. Furthermore, molecular dynamics (MD) simulations reveal that hydrogen atoms act as “low-friction brushes” between sliding layers of crystalline FeOOH, resulting also in low friction. A new model of sustainable green superlubricity is proposed. The tribo-formation of FeOOH with glycerol leads to a unique polishing process, which in turn leads to a self-sustained Elasto-Hydrodynamic Lubrication (EHL) regime until the very thin fluid film is no more than a few nanometres thick. At lower thicknesses, the hydroxide layer takes over. Wear of the ta-C coating is negligible, while wear on the steel ball is very moderate and acceptable for many practical applications, such as bio-tribology and the food industry, in which green lubrication is especially needed.

## Introduction

Friction is inevitable when two solid bodies are in contact under relative movement. Fortunately, in 1990, through calculation, Hirano and Shinjo demonstrated that static friction could entirely vanish^1^. This phenomenon was named “superlubricity”. They later fabricated a tuneable friction system by adjusting the lattice misfit angle between two contacting mica plates. The decrease of friction occurred when experimental conditions approached the superlubric state^2,3^. Superlubricity was achieved for the first time under real conditions when a molybdenum disulfide (MoS_2_) coating slid against a steel pin under ultrahigh vacuum. The investigators explained the superlubricity of bulk lamellar MoS_2_ by the formation of low-shear-strength sulfur-rich basal planes oriented in the sliding direction^4,5^. Due to the incommensurate structure, another bulk lamellar material, graphite, was reported to exhibit ultralow friction coefficient at the nanoscale while sliding against a tungsten tip under high vacuum^6^. Moreover, at the microscale, the weak van der Waals force between graphene layers makes graphene an ideal candidate for achieving superlubricity^7–9^. Graphene is also a promising solid lubricant for establishing frictionless sliding^10^.

Unlike graphite, graphene, and MoS_2_, high-vacuum conditions are not a requisite for carbon nanotubes to achieve superlubricity^11^. A high vacuum is also not required to achieve superlubricity in the liquid state, although liquids have a different definition of abnormally low friction (i.e., a friction coefficient less than 0.01). De Barros Bouchet *et al*. conducted investigations on superlubricity under boundary lubrication with environmentally friendly lubricants. They reported observing superlubricity when tetrahedral amorphous carbon (ta-C) was lubricated with an oleic acid. The formation of graphene oxide in the wear track was found to be responsible for superlubricity^12^. Additionally, they attributed abnormally low friction of the steel/steel tribo-pairs lubricated by pure glycerol to the formation of a “H-bond network” and to a water-like lubrication mechanism^13^. Furthermore, their research on ta-C/ta-C tribo-pairs sliding in glycerol revealed that the bonding of sp^2^ hybrid orbitals and OH groups is critical to the occurrence of superlubricity^14^. Luo *et al*. studied superlubricity under acidic conditions (pH ≈ 2). For the tribo-pair Si_3_N_4_/SiO_2_, superlubricity was achieved in phosphoric acid aqueous solutions^15^. The case of combining an acid with polyols, not only phosphoric acid and sulfuric acid but also boric acid, hydrochloric acid, oxalic acid, and sulfamic acid were found to result in superlubricity^16–18^. However, highly acidic conditions promote metal corrosion and limits its application in industry. Recently, Ge *et al*. reported that, under neutral conditions, mixing a weak acid (boric acid) with polyethylene glycerol (PEG) as lubricant effectively achieves superlubricity^19^. Despite the relatively large wear scar developed by corrosive effects and their tendency to shift the lubrication to the hydrodynamic regime at high speeds, this approach represents a substantial step towards industrial applications.

In the present work, to further reduce wear and a stable superlow friction coefficient in the long term, we study pure glycerol as a model green lubricant. First, lubrication tests were performed with a steel/steel configuration; a measured friction coefficient of 0.02 for this configuration has already been reported in the literature, but this low coefficient was observed to persist for a short time. By changing the steel flat to a ta-C-coated one and for the first time, we achieved superlubricity under boundary lubrication conditions (the lambda ratio defined as the ratio of oil film thickness to the composite roughness of the two surfaces is less than unity). The durability was studied by prolonging the sliding time up to 7.4 hours. To elucidate underlying mechanisms, characterization and analytical tools, such as AFM, SEM and XPS, were employed. Finally, by considering the results combined with theoretical calculations and molecular dynamic simulations, we propose a new superlubricity model based on the self-sustained thin-film EHL regime acting in conjunction with OH-terminated surfaces. A critical role is played by *in situ* chemical polishing of the contacting surface as a result of hydroxyl group surface chemical finishing of the steel by glycerol and ta-C.

## Experimental Section

### Materials

Pure glycerol (≥99.5%) was purchased from Sigma-Aldrich. AISI 52100 steel balls with a diameter of 12.7 mm and steel square flat samples with an initial roughness Ra of 2.2 nm were acquired from PSC Instruments, UK. The 3.5 μm-thick ta-C coating was produced by a PVD process (arc-ion plating) using a graphite target and was cast onto a polished bearing steel flat (dimensions 5 mm × 5 mm) with a thickness of 2 mm. The ta-C coated flats were polished by lapping with a diamond slurry and did not show any delamination excepted at the rim. Fraunhofer IWS in Dresden prepared and provided the ta-C-coated steel flats, whereas ta-C-coated steel balls (Ra ≈ 32.3 nm) were purchased from the Onward Company, Japan. The different physical, chemical and mechanical properties of the ta-C coating and the AISI 52100 bearing steel are shown in Table 1.

**Table 1:** Main properties of tribo-materials used.

	Hardness (GPa)	Elastic Modulus (GPa)	Initial roughness (nm)	C sp_3_/sp_2_ ratio (%)
steel ball	8	210	19.7	—
steel flat	8	210	2.2	—
ta-C ball	65	650	32.3	70
ta-C flat	55	650	2.7	70

### Friction experiments

The ball-on-flat sliding tests were performed using a reciprocating tribometer with a sinusoidal movement. Before the tribo-tests, both the balls and the flats were ultrasonically cleaned in heptane for 30 minutes, then in acetone for 5 minutes. At the contact area between ball and flat, 50 microliters of lubricant was supplied using a syringe. To ensure that the tribo-tests were performed under a boundary lubrication regime, the normal load was fixed at 3 N, corresponding to a maximum Hertzian contact pressure (P_max_) of 577 MPa and an average pressure of 386 MPa. Two maximum sliding speeds were used in our tests: 1 mm/s and 3 mm/s; the temperature was maintained at 50 °C for all tests. The length of the stroke was adjusted to 8 mm to ensure a large kinematic length compared with the diameter of the contacting area (approximately 0.1 mm). All tests were performed three times and demonstrated excellent repeatability. With the advantage of reciprocating movement, the accuracy of the measured friction coefficient for this system was approximately 0.002. However, a coefficient of friction (CoF) less than 0.004 is difficult to measure, and we took special care in the data processing (see Supplementary Information for further details about data processing).

### Surface analysis, microscopy and topography

Before performing surface analysis, to avoid excess glycerol affecting the analysis results and to retain the chemical bonds on the surface, samples were carefully rinsed with distilled water only. They were then dried inside a stove at 80 °C. Interferometry (Brüker Ltd., Billerica, USA) was used to obtain wear-track roughness profiles of samples and to measure the total wear volume of the ball. Digital microscopy (VHX-1000, Keyence Ltd., Osaka, Japan) and scanning electron microscopy (SEM) (MIRA3, TESCAN Ltd., Fuveau, France) were used to measure the wear diameter on the steel balls and the wear scar width on the flats. For SEM observations, the operating voltage was either 15 kV or 20 kV. Moreover, two detection modes were used to acquire SEM images: secondary electron (SE) and in-beam secondary electron (with a highly efficient detector). All images are shown at a fixed magnification of 10^3^ for comparison. XPS surface analysis was performed using an ULVAC-PHI Versa Probe II spectrometer equipped with a monochromatized Al Kα X-ray source operating at 1486.6 eV. The size of the X-ray spot is 50 μm and the energy scale was calibrated using the C1s binding energy (BE) located at 284.8 eV. Analyses were first performed recording a survey spectrum on a wide range of 1200 eV in order to identify all chemical elements. Afterwards, a scanning of the individual photo-peaks was undertaken over a smaller range of 15–25 eV to establish the different chemical states of the detected species and to perform quantitative analysis using PHI multipack software. XPS photo-peaks were fitted with a Shirley background and the quantification was calculated with Wagner sensitivity factors. Before performing XPS analyses on the tribofilms, the specimens were cleaned by several immersions in pure distilled water. All the parameters needed for the fitting such as the peak area ratio, the difference between doublets binding energies and the Full-Width at Half-Maximum (FWHM) were fixed in order to obtain the most appropriate chemical meaning. A 2 keV argon-ion beam was used for performing depth profiling and the sputtered area was 2 mm × 2 mm.

Roughness measurements of the surfaces before and after friction were carried out by atomic force microscopy (AFM, Veeco, USA) under ambient conditions. All the images were recorded in tapping mode; the scan area was 50 × 50 μm². Before friction tests, the virgin surfaces of the steel ball and the ta-C flat were also analysed.

### Method for locating the ta-C wear track in XPS

The DLC wear track was practically invisible under SEM under our working conditions. Fortunately, after the test, the wetting of the DLC surface by glycerol changed inside the wear scar. During the cleaning process to remove glycerol from the DLC-coated disk with paper, the difference in hydrophobicity inside and outside the wear scars made the wear track optically visible. Hence, an approximate location of the wear track was obtained and marked. For XPS analysis, several successive analysis spots were aligned to ensure that they crossed the location of the wear track. Therefore, both inside- and outside-wear-scar XPS spectra of ta-C flat were successfully acquired.

### MD simulation

MD simulations were performed to understand the friction behaviour of iron hydroxides formed in a steel/ta-C tribo-system lubricated with glycerol. The NEW-RYUDO program developed by Prof. Miyamoto’s group^20–23^ was used. In this program, the motion of atoms is solved by the Verlet algorithm^24^ and a long-range Coulombic interaction was computed by an Ewald method^25^. The system temperature was maintained by controlling the velocity of all atoms in a simulation cell. To express atomic and molecular interactions, we used a force field similar to that by Onodera *et al*.^23^ Notably, parameters for the force field were determined on the basis of accurate quantum chemistry calculations, such as those performed under density functional theory (DFT). The details of the parameterization procedure are described elsewhere^22^.

## Results

### Friction results

**Figure 1:**
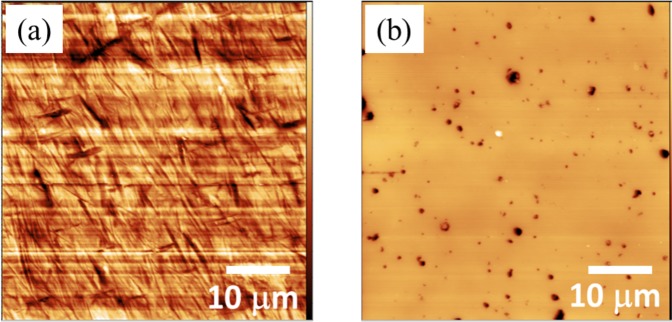
AFM images of as-received surfaces of (**a**) a steel ball and (**b**) a ta-C-coated steel flat showing a smooth surface and typical holes (in black).

The AFM images (Fig. 1) show that the initial Ra of the as-received steel ball was 19.7 nm. The steel flat has a roughness of 2.2 nm, and the ta-C-coated ball has a roughness of 32 nm. The ta-C flat surface is much smoother, with an Ra equal to 2.7 nm, and the black points correspond to holes typically found in this type of coating after carbon droplets from the deposition process have been removed by polishing. The presence of small holes compared with the contact area is not a problem for the conformal contact geometry and it has even the advantage to work as oil reservoirs. These two materials are representative of practical use in industrial applications, especially the automotive industry.

**Figure 2:**
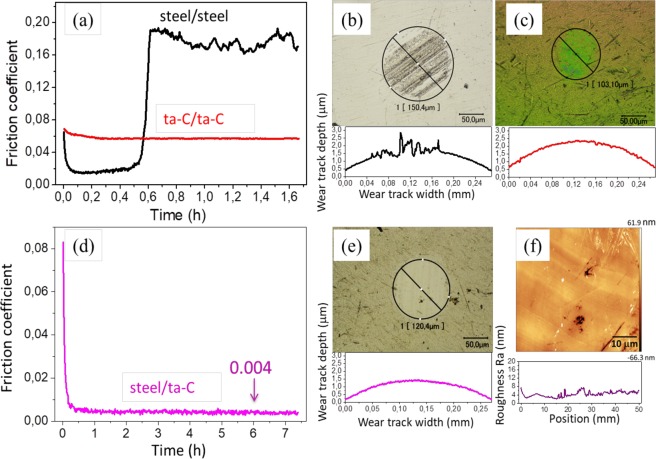
Friction curve as a function of test duration for lubrication of (**a**) steel/steel, ta-C/ta-C and (**d**) steel/ta-C tribo-pairs by glycerol at 50 °C, a sliding speed of 3 mm/s, and a P_max_ of 577 MPa. Optical images and depth profiles of wear scars on steel balls for the combinations (**b**) steel/steel, (**c**) ta-C/ta-C, and (**e**) steel/ta-C at the final stage. (**f**) AFM images corresponding to the region inside the wear scar of the steel ball after the 7.4 h-duration test. Topographic line-scans across the wear scar diameter are shown in all cases.

Figure 2(a) shows the evolution of the CoF as a function of time between self-mated steel samples in glycerol at a sliding speed of 3 mm/s and a temperature of 50 °C. The evolution of the friction can be divided in three parts: (i) starting with the running-in process (approximately 0.05 h), the CoF rapidly decreases from 0.06 to 0.016; (ii) low friction remains stable until approximately 0.25 h. The friction coefficient then slowly increases from 0.016 to 0.03 over 0.31 h. (iii) The CoF then drastically increases to 0.19 and maintains a high friction value afterwards.

The optical images in Fig. 2(b) show the aspects of worn steel surfaces. After the friction test, the steel ball showed a wear scar diameter of approximately 150 μm and was covered with many scratches; the flat surface, which also exhibited numerous scratches, showed a clear wear track. This observation was confirmed by the roughness profiles of the tribo-pair measured by interferometry. As a result of these scratches emerging, the contacting surfaces became rough and the lubricity of the liquid film was destroyed, corresponding to high friction at the end of the test. However, during the first 0.56 h, the damage on surfaces was minimal.

During the low-friction period, no measurable wear was detected on the flat and the wear volume of the ball was about 1.1 × 10^3^ μm^3^. However, at the end of the test, black scratches accumulate inside the wear scar of the ball, leading to a negative wear volume of −2.5 × 10^3^ μm^3^. As for the steel flat at the end of the test, matter is missing and the surface and wear volume is 4.4 × 10^4^ μm^3^. In a previous paper, the occurrence of low friction in steel/steel contact was attributed to a thin fluid film of glycerol easily shearing and a nanometre-thick water film forming due to the degradation of glycerol under severe conditions^13^. Therefore, the steel/steel combination lubricated by glycerol cannot provide low friction and low wear over a long period.

As for the self-mated ta-C friction pair tested in glycerol at 50 °C and 3 mm/s, the first 0.4 h of the test shows the CoF decreasing from 0.07 to 0.06. The CoF then remains stable at 0.06 until the end of the test. With respect to wear after the sliding test, no measurable wear is observed on the ta-C flat, whereas a wear diameter of 103 μm and colour change from initial yellow to green is observed (Fig. 2(c)). The colour is due to the thickness change of the optically transparent ta-C coating changing the refraction of visible light. However, wear could not be detected by interferometry. In this case, the relatively high CoF limits further applications.

Eventually, the steel flat was replaced with a ta-C-coated flat. Figure 2(d) shows the CoF of the steel ball sliding against the ta-C-coated flat under the same conditions as the self-mated steel and ta-C pairs. During the first 0.5 h running-in period, the CoF decreased sharply from 0.084 to 0.004. Thereafter, the superlow friction coefficient (0.004) persisted until the end of the test (*i.e*. 7.5 h). This amazing and unexpected result warranted further investigation. After the long-duration test, no measurable wear was observed on the ta-C flat. On the steel ball, the wear track diameter was approximately 120 μm, corresponding to a final contact pressure of 300 MPa (Fig. 2(e)). The AFM image in Fig. 2(f), which corresponds to inside the wear track on the steel ball, shows an extremely smooth surface with an Ra of 4.30 nm. Polishing occurred within the contact zone.

### Surface analysis

**Figure 3:**
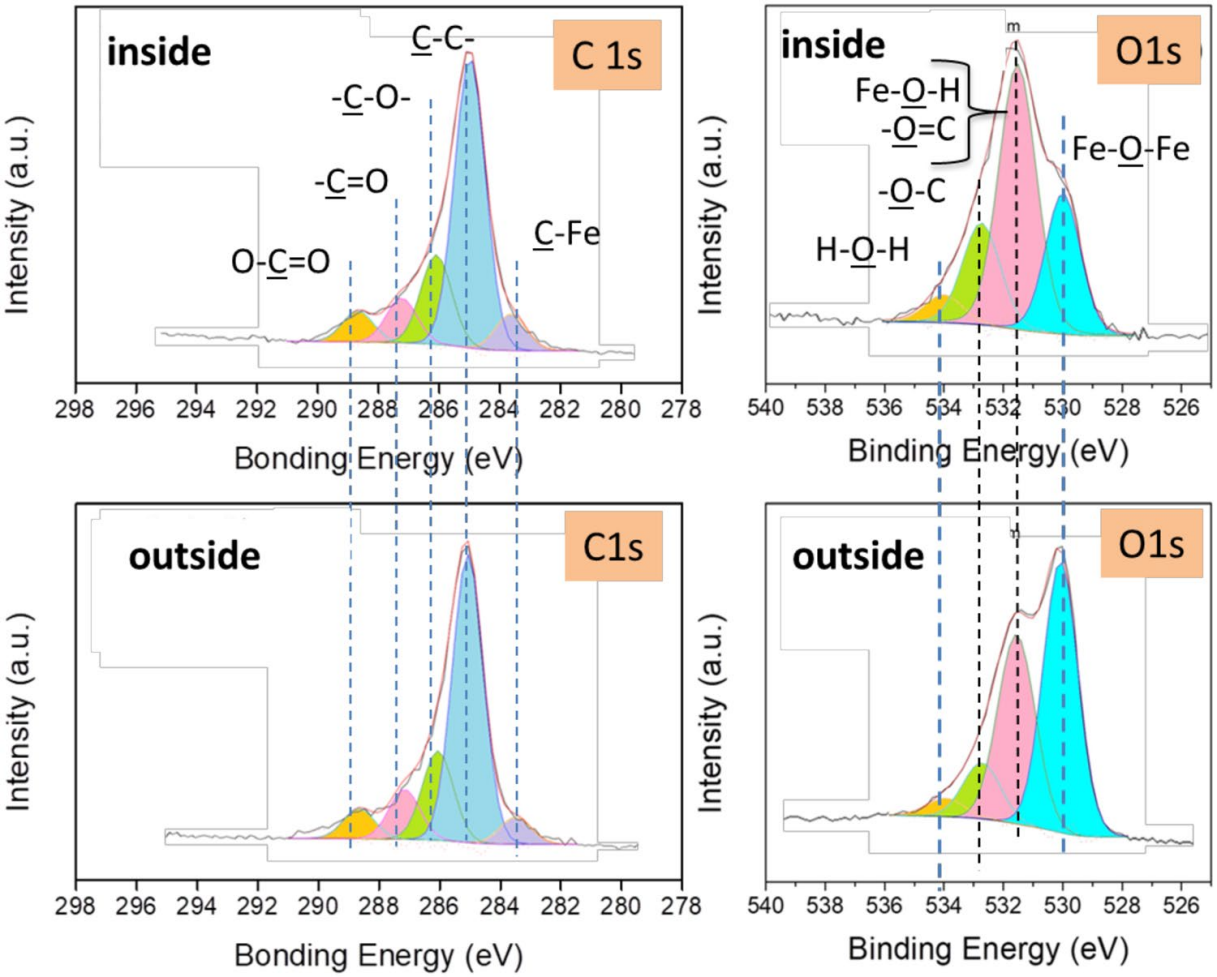
High-resolution C 1s and O 1s XPS spectra recorded on the steel ball (steel/ta-C tribo-pair slid in glycerol at 50 °C). Degradation species of glycerol (ketones, acids, etc.) are found on the steel surface, whereas more iron hydroxide is found inside the wear scar.

To study chemical reactions between the surfaces and glycerol, the regions inside and outside the wear track on the steel ball were analysed by XPS. More attention was paid to the C 1s and O 1s oxygen photopeaks in the two cases (Fig. 3 and Table 2). A characteristic peak corresponding to iron oxides is observed at a binding energy (BE in the following) of 530.0 eV, in good agreement with data from the literature. In addition, the peak at 531.5 eV BE is attributed to either an iron hydroxide or a carbonyl (O=C) bond^20^. The corresponding low intensity of the (C=O) contribution at the C1s peak at 288 eV BE confirms the predominance of hydroxides in the wear scar. Because hydroxides usually cover the top of the oxide layer, its thickness is very small, approximately 1 nm. The peak detected at 534.1 eV BE could correspond to water molecules adsorbed onto the hydroxides. This water may originate from the degradation of glycerol or the absorption of molecular water from the humid air because iron hydroxide is hydrophilic. The peak at approximately 532.8 eV BE is attributed to (C-O-) bonds present in the glycerol, with a higher contribution inside the wear track than outside. Carbonyl and carboxyl groups (C=O and O-C=O, respectively) are clearly observed both inside and outside the steel wear scar. These chemical species originate from the chemical reaction of glycerol with the oxidized steel surface at the temperature of 50° ^26^.

**Table 2:** Details on XPS binding energy of the different chemical species.

	C-Fe	C-C	C-O	C=O	O-C=O	Fe-O-Fe	Fe-O-H	O=C
Energy (eV)	283.7	285.1	286.1	287.4	288.9	530.0	531.5	532.8
FWHM (eV)	1.2	1.2	1.2	1.2	1.2	1.3	1.5	1.5

**Figure 4:**
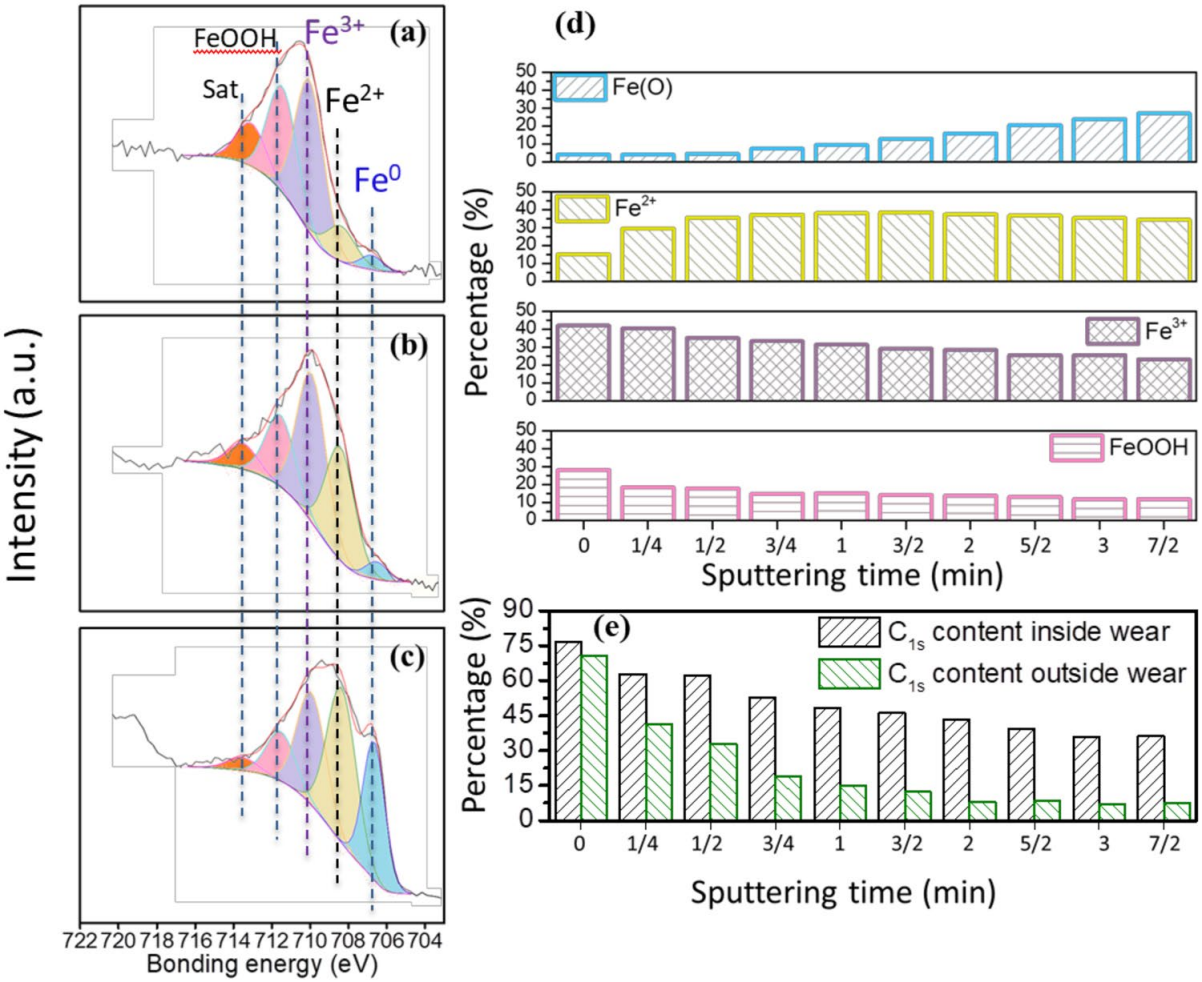
High-resolution Fe 2p_3/2_ XPS spectra after different sputtering times (take-off angle 25°) in the case of a steel ball against a ta-C flat in glycerol at 50 °C, speed 3 mm/s: (**a**) before sputtering, (**b**) after 0.25 min sputtering, (**c**) after 3.5 min sputtering. (**d**) Evolutions of the peak intensities as a function of the sputtering time. (**e**) Carbon content as the intensity ratio C 1s/(C 1s + O 1s + Fe 2p_3/2_) both inside and outside the wear track as a function of the sputtering time.

This intriguing hydroxide layer is studied in more detail in the Fe 2p_3/2_ XPS spectrum recorded at a take-off angle of 25° (Fig. 4 and Table 3). The steel surface was sputtered with argon ions for sputtering times ranging from 0.25 min to 3.5 min to obtain depth concentration profiles of the chemical species. The iron oxide already detected on the basis of its O 1s oxygen peak, can now be separated into two contributions: FeO/Fe_3_O_4_ (at 708.5 eV BE as Fe^2+^) and Fe_2_O_3_ (at 710.1 eV BE as Fe^3+^) (see Fig. 4(a)). Meanwhile, metallic iron (Fe^0^) is consistently observed at 706.9 eV BE, indicating that the tribo-film thickness is no greater than 3.5 nm. The peak at 711.7 eV BE unambiguously confirms the presence of the hydroxide on the surface^27–29^.

**Table 3:** XPS binding energy of the different chemical species corresponding to Fig. 4.

	Fe^0^	Fe^2+^	Fe^3+^	FeOOH	satellite
Energy (eV)	706.9	708.5	710.1	711.7	713.8
FWHM (eV)	1.35	1.8	1.8	1.8	1.8

The peak at 713.8 eV is a satellite peak of the Fe 2p core level and has no chemical meaning. The Fe 2p_1/2_ XPS spectrum displays the same features as expected but with three times less signal (not shown here). After 0.25 min of etching, a sharp decrease of the hydroxide peak is observed, correlated with an increase in the peak intensity of FeO and a decrease in the peak intensity of Fe_2_O_3_ (Fig. 4(b–d)). Considering the sputtering time of the native iron oxide layer of steel that is estimated to 4 nm, the hydroxide top layer is calculated to be approximately 0.5 nm thick and is covering a layer of Fe_2_O_3_. The Fe_2_O_3_ is covering a layer of FeO/Fe_3_O_4_ near the metal surface, and the FeO/Fe_3_O_4_ layer is itself covering the steel substrate. After 3.5 min of etching, all of the characteristic iron peaks remained in the spectrum, indicating that no clear line separates each layer because the layers have likely diffused into each other or have different thicknesses (Fig. 4(c)).

Apart from the evolution of the Fe 2p_3/2_ peaks with etching, the carbon content decreases continuously both inside and outside the wear scar (Fig. 4(e)). More interestingly, in both cases, the carbon content is initially greater than 70% atomic percent decreases (37% inside, 8% outside). The high carbon content before sputtering is usually caused by adventitious carbon. However, in this case, the tribo-film clearly contains a substantial amount of carbon because the high-resolution C 1s spectrum after 7/2 min sputtering shows only a small contribution content of oxidized species. Therefore, the C-C peak remains as the major contribution along the depth profile.

**Figure 5:**
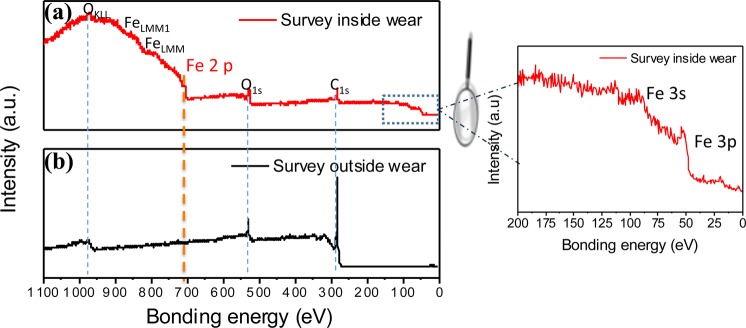
XPS survey spectra of the ta-C flat (steel/DLC tribo-pair sliding in glycerol at 50 °C, 3 mm/s). The regions inside and outside the wear scar are compared. Results clearly show that iron species were transferred from the steel substrate to the ta-C coated flat.

**Figure 6:**
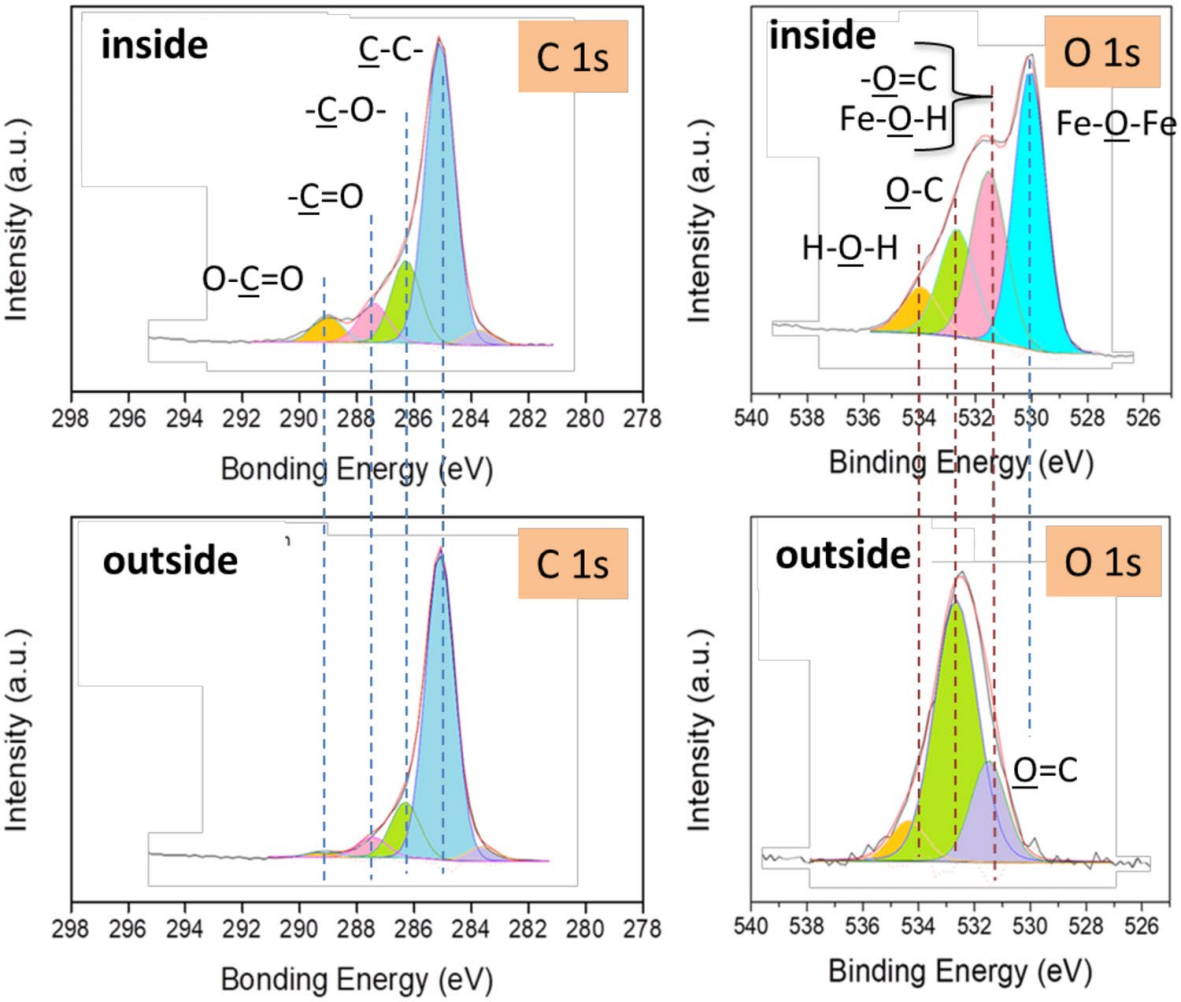
High-resolution XPS analysis of the ta-C flat (steel/DLC tribo-pair sliding in glycerol at 50 °C, 3 mm/s. The regions inside and outside the wear scar are compared. Iron oxides and hydroxides were transferred from the steel substrate onto the ta-C surface during friction.

In the following, we focus on the XPS analysis on the ta-C flat (Figs 5 and 6). In the case of the ta-C flat outside the wear track, the XPS spectrum shows two major contributions: O 1s and C 1s. However, the main difference between the spectra corresponding to inside and outside the wear track is the presence of the iron photopeaks Fe 3 s, Fe 3p and Fe 2p inside the wear scar (see Fig. 5). Since no iron peaks can be detected outside the wear track and no iron exists in pure glycerol, the iron atoms inside the wear track definitely originate from the counterpart, the steel ball. The core levels of carbon and oxygen inside and outside the wear scar on the ta-C flat are shown in Fig. 6 and Table 4.

**Table 4:** XPS semi-quantitative analysis of the different chemical species corresponding to Fig. 6.

	C-C	C-O	C=O	O-C=O	Fe-O-Fe	Fe-O-H	O-C	H-O-H	Fe_2p_
Inside (%)	35.3	9.7	4.6	3.1	16.6	11.1	7.4	3.1	7.3
Outside (%)	64	11.7	4.2	1.4	0	3.6	10.8	1.4	0

The iron oxides bond (Fe-O-Fe at 530.0 eV BE) and iron hydroxide bond (Fe-O-H at 531.5 eV BE) are significantly detected inside the ta-C wear track (in red in Table 4) whereas the C=O contribution only slightly changed. The C 1s carbon peaks comprise O-C=O peaks, and this peak is more intense in the spectrum corresponding to inside the wear scar The ketone moiety is not present in the glycerol molecule, so it is certainly formed by the tribochemical reaction on the steel side. These results give clear evidence that friction is responsible for the molecular degradation and oxidation of glycerol.

## Discussion

This study compares the behaviour of three different tribo-pairs: steel/steel, ta-C/ta-C, and steel/ta-C lubricated by glycerol at 50 °C under the boundary regime (lambda ratio <1 in all cases). Superlubricity (a CoF less than 0.01) is achieved only with the steel/ta-C material combination.

### Lubrication mechanism

The initial steel/ta-C tribo-pair has a composite roughness of 19.9 nm, and the calculated EHL film thickness is 4.7 nm (see Supplementary Information). The calculated initial maximum Hertzian contact pressure is 577 MPa, and the corresponding mean pressure is 382 MPa. Therefore, lubrication is dominated by interactions between asperities (the so-called boundary regime) so that initially, the role of the viscous fluid is practically negligible. However, the steel surface becomes progressively more and more polished in the presence of glycerol and the wear scar diameter caused by chemical wear increases approximately to 120 μm at the end of the test, compared with a theoretical Hertzian diameter of 100 μm under the initial static conditions. The increase of the surface conformity in the contact reduces the mean contact pressure from 382 MPa to 300 MPa, and the surface roughness decreases to 4.3 nm, as measured by AFM. The resulting effect is a sharp decrease of the CoF to 0.004 within 0.03 h. As a consequence, the polishing effect results in a lambda value slightly greater than 1 and the thin glycerol film will start carrying a substantial part of the load in the EHL regime. If the average pressure is assumed to act over the entire contact disc, then the average viscosity according to Barus is 4.2 × 0.142 = 0.59 Pa∙s.

If a constant gap of 4.7 nm and a velocity difference of 3 mm/s are assumed, the shear rate is 6.4 e5 s^−1^. Computing the shear force only inside the Hertzian contact (a = 52 μm) reveals that Ff = 3.2 mN; thus, a friction coefficient of 0.0011 is found. This low value does not account for shear forces outside the contact or for solid-solid interaction; hence, a slightly higher friction coefficient is to be expected. The experimental value of 0.004 is therefore in good agreement with nm-thick film EHL regime at work. Interestingly, nearly the same superlow friction values were calculated for the two other cases (steel/steel and ta-C/ta-C pairs); however, polishing did not occur and the friction data in Fig. 2 show that low friction is not sustained in these cases.

Such superlow values have already been obtained for highly polished steel/steel contact under rolling/sliding conditions, which are much less severe than pure sliding conditions. The film thickness was of the same order as ours^21^; in our case, however, the EHL regime was self-sustained in pure sliding. Notably, the literature includes several examples in which an increase in the wear scar diameter resulting from huge chemical wear at high speeds correlates with a drastic collapse of the contact pressure to a few tens of MPa. This situation effectively leads to friction values smaller than 0.01, which are classically explained by hydrodynamic lubrication; see, for example, ceramics in water^30,31^. Our results are completely different because the pressure does not collapse at all and the contact remains in the EHL and/or mixed regime at low speeds so that sample wear is very moderate and lubrication is suitable for practical use.

To investigate whether a fluid film plays a substantial role in achieving superlubricity, we used two different sliding speeds. The combination of 50 °C and 3 mm/s gave a fluid film thickness of 4.7 nm and a theoretical CoF of 0.0011 (the fluid film thickness calculation method is detailed in the Supplementary Information). The decrease of sliding speed (1 mm/s at 50 °C) results in a decrease in film thickness to 2.3 nm, which is substantially less than the composite roughness (19.9 nm); thus, severe boundary conditions occur. The friction coefficient was measured as 0.01 on the Stribeck curve, which is a very low value for these conditions (see Fig. S1 in Supplementary Information). This value can be compared with a theoretical CoF of approximately 0.001. This result shows that a suitable fluid film thickness is necessary to achieve superlubricity. The CoF of approximately 0.01 under severe boundary conditions is not explained by EHL; another mechanism is at work.

### Polishing mechanism

Because self-polishing represents one of the key roles in the superlow-friction process, we performed additional experiments by varying the test duration (by exchanging the friction pair with a new one for each test).

**Figure 7:**
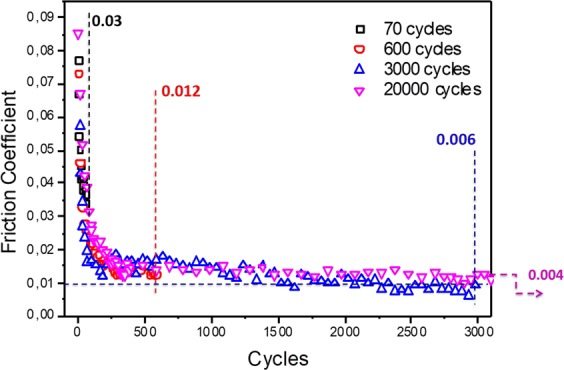
Evolution of the friction coefficient against time for a steel/ta-C contact lubricated by glycerol at 50 °C, speed 3 mm/s. Several tests were performed with various numbers of cycles (70, 600, 3000 and 20,000). Each test was run with new samples. All of the friction curves are practically superposed. Note that the 20,000 cycles test is not shown till the end for clarity.

Friction results are shown in Fig. 7 for 70, 600, 3000 and 20,000 cycles, respectively. Note that the results of the 20,000 cycles test are not shown to the completion of the test to emphasize the beginning of the polishing mechanism. First, the different friction curves are well aligned with each other in a master curve, showing that the polishing mechanism is reproducible. The process shows three steps: From zero to 70 cycles, the CoF decreases from approximately 0.1 to 0.03. From 70 to 600 cycles, the CoF decreases until reaching a plateau at 0.015. Finally, from 1000 cycles to 20,000 cycles, the CoF decreases progressively to values less than 0.01.

**Figure 8:**
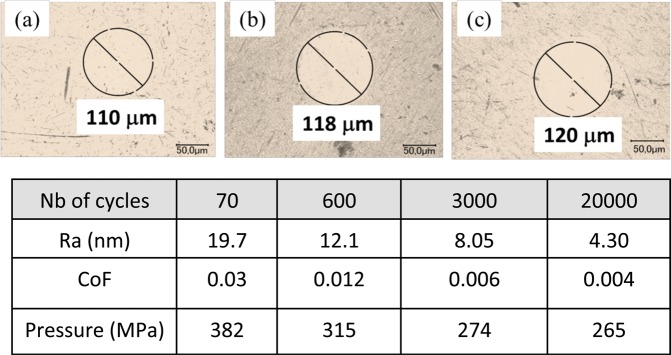
Optical images of wear scars on steel balls corresponding to different numbers of cycles: (**a**) 600, (**b**) 3000 and (**c**) 20,000 cycles are demonstrated. The wear scar at 70 cycles was not visible; therefore, the mean pressure was that calculated on the basis of Hertz theory.

The effect of polishing is hardly visible in the SEM image of the wear scar on the steel ball; however, it is visible on the optical image and roughness profiles measured by interferometry (Fig. 8). At fewer than 600 cycles, there is no wear of the ball; wear occurs between 600 and 3000 cycles, but the wear volume remains very small. At the end of each test, the roughness was quantitatively measured inside the wear scar by AFM, and the Ra values were compared with the average CoF values (see Table 5). At 70 cycles, the wear scar on the ball was not visible; we therefore reported the Ra of the virgin steel surface outside the wear scar. The correlation between the Ra and the CoF is clear, and the data show that the friction decreases with decreasing roughness. Figure 8 also shows the evolution of the mean contact pressure during polishing. The pressure stabilizes at approximately 300 MPa, which is, of course, not consistent with a hydrodynamic lubrication mechanism, as often claimed by authors in similar papers on liquid superlubricity. These experiments demonstrate that polishing is closely linked to low roughness and therefore to low friction.

**Table 5:** Evolution of friction coefficient and mean contact pressure as a function of the number of cycles.

Nb of cycles	70	600	3000	20000
Ra (nm)	19.7	12.1	8.05	4.30
Friction coefficient	0.03	0.012	0.006	0.004
Pressure (MPa)	382	315	274	265

The polishing level is represented by the average roughness (Ra) determined by AFM as well as by the apparent contact pressure calculated from the scar diameter for each case.

We subsequently investigated the mechanism of polishing in greater detail. An experiment was first performed to generate a rough surface with large wear scratches on the contact surfaces by sliding a self-mated steel tribo-pair in glycerol under 50 °C at 3 mm/s. Afterwards, a new friction test was carried out under the same conditions but with the steel flat replaced with a ta-C flat to investigate whether wear marks could be eliminated by polishing. In this test, the steel ball was not changed to ensure that the contact area was located at the same location on the ball.

**Figure 9:**
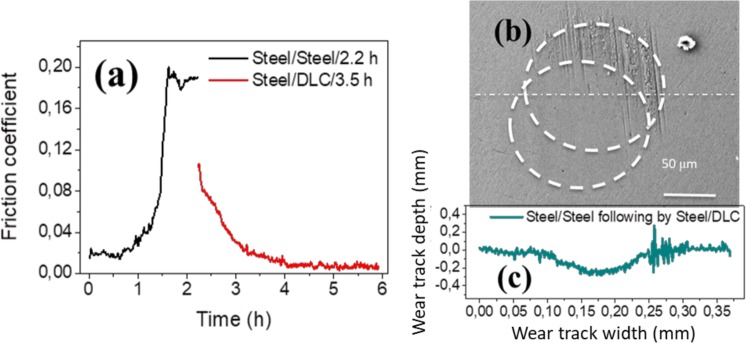
(**a**) Friction coefficient as a function of test duration, as determined by first sliding steel against steel in glycerol for the first 2.2 h followed by sliding the same part of the steel ball on a ta-C disc in glycerol at 50 °C and 3 mm/s. (**b**) SEM image of the wear track on the steel ball after the test; the images shows the polishing mechanism and the two wear scars superimposed. (**c**) Corresponding depth profile in the wear track. Since wear is limited, ball shape depth profile as Fig. 2(e) is not sufficiently clear to demonstrate the depth change after friction. The depth profile was standardized from ball to plane. Negative wear track depth values indicate missing material.

The results are shown in Fig. 9. When a self-mated steel disk and ball are slid against each other, the friction coefficient increases from 0.02 to greater than 0.16, accompanied by the appearance of a large number of scratches on the surface (Fig. 2(a)). When the steel disk is exchanged for a ta-C one, the friction coefficient first decreases suddenly to 0.11 and begins continuously decreasing until 0.004 after 4 h of testing, then remains stable thereafter. The SEM image in Fig. 9(b) shows the wear scars on the ball at the end. The 172 μm diameter wear track is partially covered with large scratches. A 115 μm diameter scar with very smooth surface is superimposed onto the previous one when the ta-C flat is used. Fortunately, the two wear scars are not exactly at the same place; thus, they can be observed together. This evidence clearly shows the occurrence of a polishing mechanism that increases the lambda ratio by decreasing the roughness.

### Iron hydroxide formation and MD simulations

Our XPS results corresponding to the friction process show that iron oxides interact chemically with the glycerol molecules and that the reaction produces iron hydroxide on both the ball and the flat. A possible reaction is as follows:

$${{\rm{Fe}}}_{{\rm{x}}}{{\rm{O}}}_{{\rm{y}}}+{{\rm{C}}}_{3}{{\rm{H}}}_{8}{{\rm{O}}}_{3}\to {\rm{Fe}}{({\rm{OH}})}_{{\rm{z}}}+{\rm{Cs}}+{{\rm{H}}}_{2}{\rm{O}}$$

We propose here that this reaction produces iron oxyhydroxide, FeOOH (lepidocrite), which is preferred to the hydrated form, Fe(OH)_3_, because of its 2-D lamellar structure in the contact configuration. The tribochemical reaction also generates carbon and water. Solid carbon and liquid water are preferentially formed rather than gases (carbon monoxide (CO) and hydrogen gases, for example) because of the antagonist effect of the high contact pressure, which prevents gas formation in the tribochemical reaction (according to Le Châtelier’s principle). This effect could explain why a relatively high carbon content is found by XPS even after depth profiling inside the wear track. Moreover, the low friction generated by the OH-terminated groups was highlighted in the last decade^14^. Recently, metal hydroxides have been reported to be the key factor to achieve superlubricity of Nitinol/steel contact lubricated by castor oil^32^.

To understand the friction behaviour of FeOOH, we conducted a MD simulation for its slab model. First, the structure of the model compound FeOOH was reproduced to validate the method (see Fig. S2 in the Supplementary Information). The MD-obtained structures before and after sliding are depicted in Fig. 9. Through the MD simulation, sliding between hydrogenated surfaces is clearly observed.

**Figure 10:**
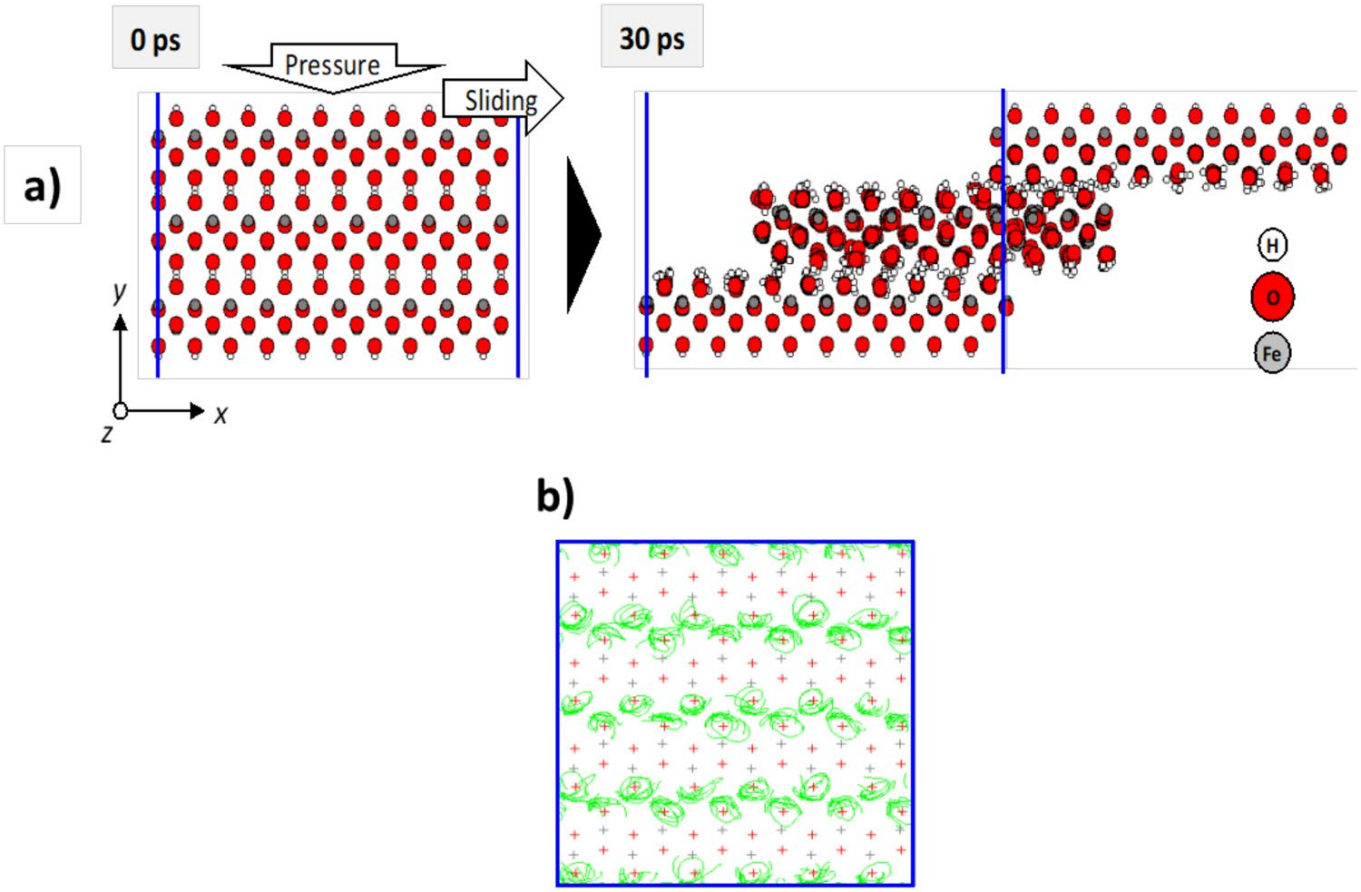
(**a**) Results of MD simulations for gamma-FeOOH under friction conditions. In the simulation, constant pressure in the y direction was applied to the topmost surface as it slid with constant velocity in the x direction. The pressure was 1 GPa. The sliding speed was set as 100 m/s to demonstrate a friction effect within a reasonable computation time. The bottom layer of the model was fixed. The temperature was maintained at 300 K. The integration time was set as 0.1 fs, and a non-equilibrium MD simulation by the NEW-RYUDO program was performed for 500,000 steps. Although a periodic boundary was applied in the simulation, it is not shown here to clarify the motions of atoms. (**b**) Trajectories of H atoms during sliding simulation showing specific rotation movements.

This self-mated FeOOH system shows a low friction in commensurate structural configuration (calculated CoF 0.06). The motion of interlayer hydrogen atoms can explain the mechanism for this low friction. The trajectory of hydrogen atoms during MD simulation is shown in Fig. 10b. It can be observed that hydrogen atoms clearly rotate in all directions and are hardly interacting with other hydrogen atoms. A quantum chemistry calculation for gamma-FeOOH shows no overlap of electron clouds between hydrogen-faced surfaces (not shown here). Accordingly, these results suggest that the origin of the low friction between FeOOH crystals is due to very weak interaction between hydrogen-faced layers. Hydrogen plays a role as ‘low friction brush’ between layers of crystalline FeOOH. Unlike FeOOH, other iron oxides (FeO, Fe_2_O_3_, and Fe_3_O_4_) typically show very high friction. The calculated CoF is higher than our measurement (CoF approximately 0.01). This can be explained because the FeOOH surfaces are commensurate in our simulation, it is probable that the friction decreases when incommensurability occurs and it certainly occurs in the contact area.

**Figure 11:**
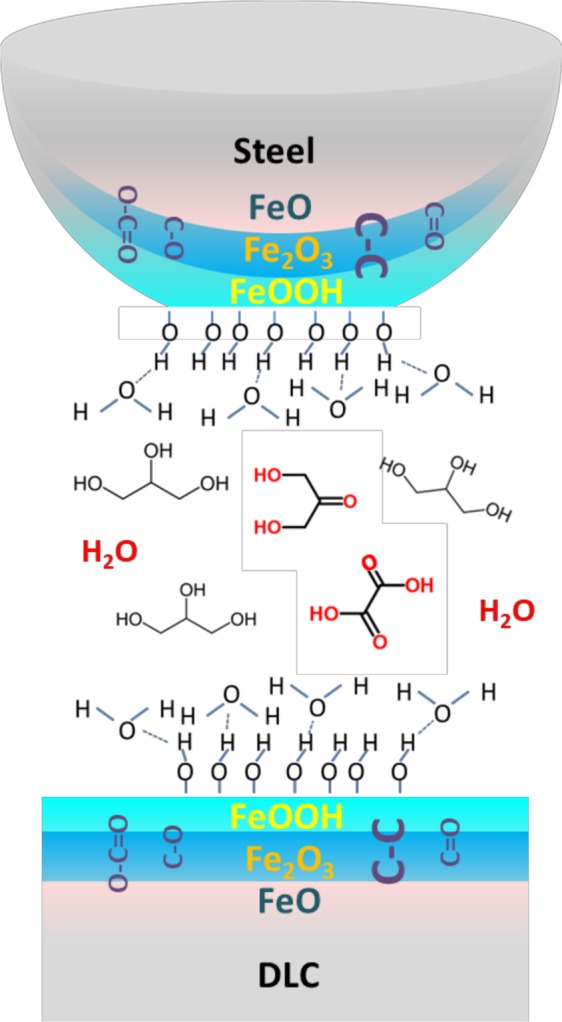
Schematic of the mechanism of superlubricity of a steel/ta-C tribo-pair in glycerol. The oxide surface is covered with iron hydroxide, giving OH termination, and a nanometre-thick fluid film ensures the EHL regime.

Accounting for all the necessary conditions to achieve superlubricity, a schematic representation of the interface is proposed (Fig. 11). The steel ball achieves an extremely smooth polished surface inside the contact zone. On top of this polished surface, a layer of FeOOH with a thickness of approximately 0.5 nm is generated by tribochemical reactions. Because of the hydrophobicity of FeOOH, water is absorbed onto the surfaces and forms a double-hydroxyl-terminated surface^33^. Between these double-water-terminated surfaces, a fluid layer composed of glycerol and possibly some of its main degradation products (e.g., water, dihydroxy-acetone, and hydroxyl-pyruvic acid) separate the contacting surfaces in the thin-film EHL regime^34^. The carbonyl and alcohol groups exist in all of the layers (FeOOH layer and iron oxides).

Carbon is also formed in the process and is found mixed with the surface species. This scheme emphasizes the three necessary conditions to achieve superlubricity: (i) gentle polishing of the steel surface in the contact area, (ii) the presence of a FeOOH layer and (iii) the presence of a thin, easily sheared EHL fluid film. The fluid film provides superlubricity, and the hydroxide layer reduces friction during solid contact and also participates in chemical polishing, possibly resulting in slip at the wall. Once the superlow regime is reached, chemical wear stops, typically corresponding to a self-sustained process. During this step, the mean contact pressure has been reduced from 386 MPa to 300 MPa, a reasonable and acceptable value and it remains stable till the end of the test duration. Moreover, as shown by the MD simulations, the unique low-shear property of lamellar FeOOH can also take over, promoting lower friction (CoF of 0.01) when occasional solid contact occurs at very low speeds.

## Supplementary information

Superlubricity of glycerol by self-sustained chemical polishing
