# Supplementary material for: Superlubricity of glycerol by self-sustained chemical polishing
Source: Sci Rep. 2019 Apr 18;9:6286. doi: 10.1038/s41598-019-42730-9 (PMC6472501; doi:10.1038/s41598-019-42730-9)
Supplement: Supplementary file 1 — Superlubricity of glycerol by self-sustained chemical polishing [file 41598_2019_42730_MOESM1_ESM.pdf]

Superlubricity of glycerol by self-sustained chemical polishing

Yun Long†, Maria-Isabel De Barros Bouchet†, Ton Lubrechtξ, Tasuku Onoderaζ and Jean Michel Martin†

† Université de Lyon, Ecole Centrale de Lyon, LTDS CNRS 5513, 69134 Ecully, France.

ξ Université de Lyon, INSA de Lyon, LaMCoS, CNRS 5259, Villeurbanne F69621, France.

ζ Advanced Materials & Process Research Department, Center for Technology Innovation – Materials, Research & Development Group, Hitachi, Ltd., 7-1-1 Omika, Hitachi 319-1292, Japan

[*] Corresponding authors.

Email; jean-michel.martin@ec-lyon.fr (Jean Michel Martin)

1. Film thickness calculation

Since the geometry is ball on flat, the Hamrock Dowson EHL film prediction should be accurate.[1] However, as the piezo-viscosity is low, the contact operates close to the iso-viscous elastic (I.E.) regime. Hence it is safer to rely on the Moes-Venner model.[ii] The film thickness (h_c) is defined as:

$$h_c = R_x * \sqrt{U} * H_c^M$$

Here R_x is the reduced radius of curvature in x direction. In our case R_x, is the radius of the ball: 6.35 mm.

U is the dimensionless speed parameter. It can be written as $U = h_0 * (m_1 + m_2) /(E' * R_x)$.

h_0 is the dynamic viscosity; m_1, m_2 are the velocity of lower and upper surface respectively. Here $m_1 = 0$ m/s and $m_2 =$ 0.003 m/s. *E'* is the reduced modulus of elasticity. *E'* is given as: $2/E' = (1 - n_1^2) / E_1 + (1 - n_2^2) / E_2$. Thus, the *n, E* are the property of the bulk material instead of the coating. Here, $E_1 = E_2 =$ 210 GPa, $n_1 = n_2 =$ 0.3 GPa. The n_1, n_2 are the Poisson ratios of the ball and the flat. $E_{1,}$, E_2 represent the modulus of elasticity of the tribo-pair.

M2=…, L=…, which is indeed close to the I.E. lubrication regime.

H_c^M is given by:

$$H_c^M = \{[(1.70t\, M_2^{-\frac{1}{9}} L^{\frac{3}{4}})^r + (1.96\, M_2^{-\frac{1}{9}})^r]^{s/r} + (47.3 M_2^{-2})^s\}^{1/s}$$

Where $r = e^{[1-6/(L+8)]}$, L is the dimensionless material parameter: $L = aE'U^{1/4}$. a is viscosity-pressure coefficients. w is the normal force.

$s = 12 - 10\ exp(-M_2^{-2})$. M_2 is the 2d dimensionless load parameter: $M_2 = w / (E'R_X^2 U^{3/4})$.

$t = 1 - exp(-0.9M_2^{1/6}/L^{1/6})$.

Taking the viscocity and a values in table 1, the fluid thickness is calculated as 4.7 nm in 50°C, 3mm/s and 2.3 nm in 50°C, 1mm/s.

Table 1. viscosity and a values on the function of temperature.[2,3]

Temperature (°C)	h_0 (mPa·s)	a
50	142	5.7
80	31.9	4.8

a values are not listed at 50°C in reference 25. We choose a value between 30°C and 60°C.

2. Data processing for superlow friction measurement

The measurement of very low friction coefficient is not straightforward and this is particularly the case when the CoF is below 0.01. Very often, this point is not discussed in detail in the literature when dealing with superlubricity experiments. Here, we take advantage of the reciprocating movement of the friction machine to easily calculate the CoF at each cycle by avoiding the position of zero line (as for rotating tribometer for example). However, there is a drawback that is the change in sliding direction at the end of stroke and the speed varying linearly between zero and maximum speed at the middle of the track (here 3 mm/s). To get rid of that, the triboscopic imaging technique is used. In Figure S1 we show data recording and processing for a steel/ta-C test with glycerol, max speed 3 mm/s, track length 2 mm. In the image, we have the force signal data at every position in the track and at every cycle in the test. For measuring the friction coefficient against number of cycles, we used retrospective analysis of triboscopic images and only data near the center of the

stroke length is extracted Figure S1 a), thus eliminating values of lower speeds and the change in direction. One dot on the friction curve corresponds to calculating friction force value over one cycle. The next point is obtained on a further cycle, one point every 300 cycles is displayed in our case (Figure S1b). The Figure S1c) also shows the real signal of friction force coming from the sensor over one cycle at different friction times (70, 600 and 20,000 cycles). It is easy to show that friction almost vanishes at 20000 cycles although average CoF accurately calculated on one cycle is 0.004 with uncertainty of 0.001. Eventually the evolution of CoF as a function of speed (the so-called Stribeck curve) is shown in Figure S1d showing accurately that superlow friction (0.01) is conserved even at the change of sliding direction and at very low speed.

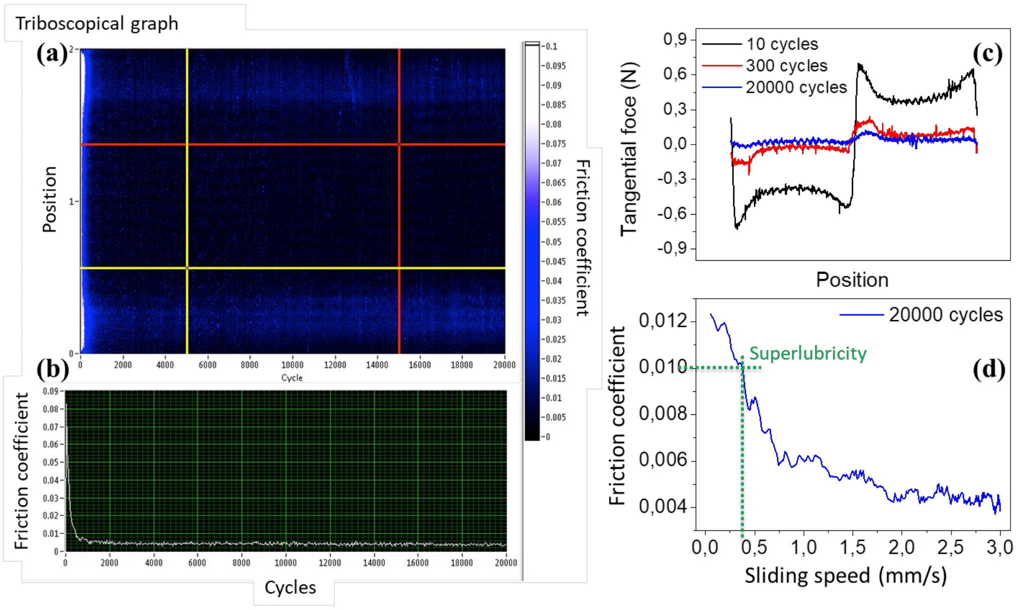

Figure S1: (a) Triboscopic image of a steel/ta-C contact lubricated by glycerol at 3 mm/s, 50 °C. b) Extraction of the average CoF as a function of the number of cycles. Only values near the maximum speed are used. c) Evolution of friction force over one cycle at different durations (10, 300 and 20,000 cycles). d) Stribeck curve over one cycle (number 20,000) showing that the CoF is below 0.01 at all speeds.

3. Computer simulation of FeOOH

To confirm the low friction of FeOOH, MD simulation has been performed. In this study, gamma-type crystalline was modeled as a representative structure of FeOOH (figure S2 (a)), which was constructed by referring X-ray diffraction experiments[X7-X8]. In the figure (b), MD-obtained structure is shown. It can be seen that all atoms are maintaining their original position, meaning that the applied force field is able to

reproduce crystal structure of FeOOH. Less change in mass density also tells us this fact.

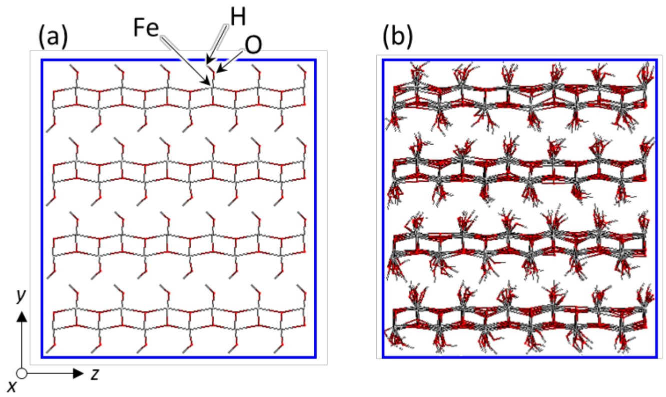

Figure S2: (a) MD simulation model for gamma-FeOOH (Fe 480 atoms, O 960 atoms, and H 480 atoms). Initial size of the simulation cell was x = 3.07 nm, y = 2.51 nm , and z = 2.32 nm. In this MD simulation, NPT ensemble and periodic boundary condition were applied. Temperature and pressure were kept at 300 K and 0.1 MPa, respectively. Integration time was set as 0.1 fs, and MD simulation by NEW-RYUDO program was done for 500,000 steps. (b) Final structure of the model. Calculated density was 3.95 g/cm^3 that is fairly close to the initial value of 3.96 g/cm^3.

References

(1) Hamrock, B. J.; & Dowson, D. Isothermal elastohydrodynamic lubrication of point contacts: Part 1—Theoretical formulation. *J. Lubr. Technol.* **1976**, 98(2), 223-228.

(2) Segur, J. B.; Oberstar, H. E. Viscosity of glycerol and its aqueous solutions. *Ind. Eng. Chem.* **1951**, 43(9), 2117-2120.

(3) Stachowiak, G.; Batchelor, A. W. Engineering tribology. Butterworth-Heinemann. **2013**.
